# Health, Well-Being, Work Ability and Work Conditions Among EAP Non-Users in Canada and the U.S.: A Quantitative Cross-National Comparison Study

**DOI:** 10.1177/11786329261471267

**Published:** 2026-07-30

**Authors:** Hong Ki Chloe Lau, Javier Mencia-Ledo, Kishana Balakrishnar, Sana Siddiqui, Raihana Premji, Paula Allen, Marilyn Grand'Maison, Allison Kelly, Ali Bani-Fatemi, Behdin Nowrouzi-Kia

**Affiliations:** 1 Department of Occupational Science and Occupational Therapy, Temerty Faculty of Medicine, 12366 University of Toronto, Toronto, ON, Canada; 2 19335 Research, TELUS Health, Toronto, ON, Canada; 3 Krembil Research Institute, University Health Network, Toronto, ON, Canada; 4 Institute for Mental Health Policy Research, Center for Addiction and Mental Health, Toronto, ON, Canada

**Keywords:** employee assistance program, occupational health, well-being, work ability, Canada, United States

## Abstract

**Background:**

Research on Employee Assistance Program (EAP) effectiveness has expanded in recent years, but comparative research across countries on people who have never used these services remains limited. Also, few studies have examined how low EAP usage relates to broader workplace factors and well-being.

**Objectives:**

The present study aims to examine cross-national differences in health, well-being, and work conditions among working adults in Canada and the U.S. who have never used EAPs. Specifically, it explores (1) satisfaction with healthcare, work ability, and job demands; (2) quality of life; (3) self-reported happiness; and (4) key demographic and occupational factors.

**Methods:**

This study employed a cross-sectional quantitative design, with adaptation from the international classification of function (ICF) framework. Data were collected through an 87-item online survey, including questions regarding demographic information and validated measures of psychological well-being (OHQ), quality of life (WHOQOL-BREF), and work ability (WAI). Participants included 179 employed adults (Canada: n = 78; U.S.: n = 101) with no prior experience of using EAP services. Data were analyzed using descriptive statistics, standardized between-country comparisons, exploratory subgroup analyses, and linear regression models.

**Results:**

No significant national differences were found in overall quality of life or happiness. U.S. participants reported significantly higher overtime hours, while other between-country differences in income, work ability, physical health, and environmental quality of life were generally small or non-significant at the overall country level. Subgroup analyses revealed that socioeconomic factors (e.g., income, education) were stronger predictors of well-being in the U.S. while occupational factors (e.g., work ability, job demands) were more influential in Canada.

**Conclusion:**

Cultural, economic, and structural differences between Canada and the U.S. shape how work and well-being interact across populations. Although overall well-being scores were similar between the two countries, differences emerged in predictors of well-being. These findings highlight the need for EAPs to adopt tailored strategies that address the specific needs of vulnerable subgroups within each national context.

## 1. Introduction

Employee Assistance Programs (EAPs) are confidential workplace-based interventions designed to help workers manage personal and work-related challenges that may affect their work performance or well-being.^1^ These programs offer a wide range of services, including counseling for emotional distress, support for job-related stress, and assistance with issues such as childcare, eldercare, harassment, substance use, and financial and legal concerns. EAPs have demonstrated efficacy in improving occupational outcomes.^2-4^ For instance, meta-analytic and quasi-experimental studies have linked EAP utilization to greater organizational commitment, increased job satisfaction, enhanced perceived support, and reduced symptoms of depression, anxiety, and work-related stress.^2-4^

Despite well-documented benefits, EAP utilization remains low across North America. In Canada, only one-third of workers report having access to EAPs,^5^ with average utilization rates as low as 9.2%.^6^ Likewise, in the U.S., only 53% of workers report EAP availability,^7^ however, utilization is even lower, averaging just 5.5%.^8^ Barriers to utilization differ slightly between the two countries. Canadian workers often cite lack of awareness (35%), doubts about effectiveness (25%), confidentiality concerns (23%), and preference for external practitioners (23%) as the main barriers.^5^ In contrast, U.S. public health workers during the COVID-19 pandemic identified access difficulties (53.1%), reliance on external providers (21.5%), and lack of awareness or motivation (11.3%) as the main barriers.^9^

These low utilization rates are particularly striking when viewed through the lens of the contrasting healthcare systems of Canada and the U.S. In Canada, the healthcare system operates under a publicly funded, universal healthcare model that guarantees access to physician and hospital services through federal and provincial taxation.^10,11^ While some services (e.g., dental, vision, outpatient prescriptions, and therapy) fall outside of this coverage, Canadians are not required to pay directly for most core healthcare services.^12,13^ Conversely, the healthcare system in the U.S. primarily relies on a private, employer-based insurance model, supplemented by public programs like Medicare and Medicaid for eligible populations.^13-15^ This fragmented system leaves nearly 9% of Americans uninsured.^16^ In fact, among those ages 18-64, 14.4% of Americans have unmet healthcare needs, primarily due to cost.^10^

Despite these structural differences, overall health outcomes in Canada and the U.S. remain relatively similar,^17,18^ suggesting that underlying factors, such as working conditions and psychological resources, may play a critical role beyond healthcare access alone. Indeed, both countries report disproportionately high levels of workplace stress, with 57% of workers experiencing daily stress, well above the global average of 43%.^17^ Additionally, workers in both nations report frequent exposure to high-speed work environments,^18^ which may increase their susceptibility to stress and burnout.^19,20^ One in five Canadians experiences a mental health issue annually, with a disproportionate burden among the employed.^21^ Comparatively, 76% of American workers report at least one symptom of mental illness.^22^

Workplace health outcomes may ultimately shape workers’ overall well-being, influencing both their happiness and quality of life. Happiness is typically understood as the presence of frequent positive emotions, life satisfaction, and a relative absence of negative affect.^23^ Quality of life is defined by the World Health Organization as “an individual’s perception of their position in life in the context of the culture and value systems in which they live and in relation to their goals, expectations, standards and concerns.”^24^ Research has demonstrated that both happiness and quality of life play critical roles in enhancing occupational outcomes such as job satisfaction,^25^ organizational commitment,^25^ and productivity,^26-28^ highlighting their importance for workers.

Importantly, EAP underutilization should not be interpreted as a uniform construct. Individuals who do not access EAP services may represent a heterogeneous group, including those who face structural or perceptual barriers, as well as individuals who may not require such services due to higher levels of resilience or other coping methods. Underutilization of EAP may therefore be a combination of unmet need, personal coping capacity, and systemic barriers. In this study, EAP non-users are assumed to capture this diversity, rather than a single underlying explanation.

While structural differences between Canada and the United States provide important contextual background, this study is not designed to reveal causal relationships. These comparisons are intended to highlight potential associations and contextual patterns that may contribute to differences in well-being across populations.

### 1.1. Present Study

Despite growing interest in the effectiveness of EAPs, there is a notable lack of comparative research that focuses specifically on individuals who have never utilized these services, particularly in cross-national contexts. Additionally, few studies have explored the relationship between the underutilization of EAP and broader workplace experiences, such as job demands, health satisfaction, and overall well-being. To address this knowledge gap, the current study investigates well-being outcomes among working adults in Canada and the U.S. who have never used EAPs. Specifically, we compare (1) satisfaction with healthcare access, work ability, and perceived job demands; (2) overall quality of life; (3) self-reported happiness levels; and (4) demographic and occupational characteristics such as income and work hours. We also explore within-country predictors of well-being to identify population-specific patterns that may inform targeted EAP outreach and development.

## 2. Methods

### 2.1. Conceptual Framework

**Figure 1.**
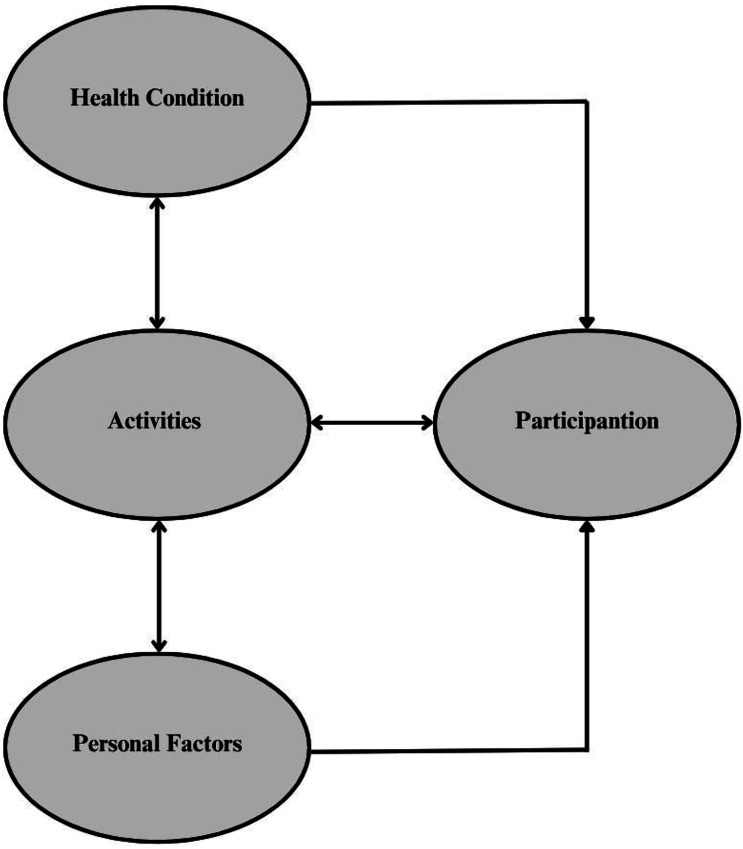
Conceptual Framework Based on WHO’s ICF framework

The present study’s conceptual framework is based on the International Classification of Functioning, Disability, and Health (ICF) framework, created by the World Health Organization (WHO). The ICF measures health and disability at the individual and population levels, including environmental factors that can occur in a given context.^25^ For the context of this paper, we were interested in health conditions in relation to quality of life and happiness, personal factors in relation to demographic characteristics, and activities and participation in relation to occupational characteristics. Figure 1 visualizes our conceptual framework.

### 2.2. Study Design

A quantitative, descriptive cross-sectional design was adopted to examine well-being and work conditions among workers who have never accessed EAP services in Canada and the U.S. This study was approved by the University of Toronto’s Ethics Review Board (Human Protocol Number: 42260) and is renewed annually. In accordance with ethical guidelines, participants’ confidentiality and privacy were protected. An invitation to participate, outlining confidentiality measures and study procedures, was provided during the initial screening process via email and surveys. Participants were required to provide informed consent by submitting an electronic signature with confirmation that they had read and understood all components of the invitation. They were also informed that they could withdraw from the study at any point during data collection without penalty. The self-administered questionnaire took approximately 25 minutes to complete and was administered through REDCap, with the results stored on the University’s servers. The study was reported in accordance with the STROBE guidelines, which serve as a framework using the STrengthening the Reporting of OBservational studies in Epidemiology (STROBE) checklist.^25^ The completed checklist can be found in Supplemental material 1. The study commenced in March 2024 and was completed in June 2025.

### 2.3. Study Participants

A total of 265 participants enrolled in the survey, with 179 ultimately completing the questions, resulting in a 68% completion rate. The rest of the participants either did not complete any questions beyond basic demographic questions or did not provide their signature to consent. The inclusion criteria required participants to be English-speaking working adults aged 18 to 65, residing in Canada or the U.S., currently employed either full-time or part-time, and with no prior experience of using EAP services. Individuals who were not employed at the time of the study were excluded. Potential participants were identified by a third-party vendor, Decision Point Research, which is “one of the largest research companies in Canada.”^26^ Their approach to recruitment involves a dedicated call center in Canada, specialized panels, including an ethnic panel and a team consisting of multilingual staff. Participants were provided with an incentive structure. We used a screening questionnaire that was administered to each potential participant to ensure they met the inclusion criteria of the study. Moreover, each participant met with a researcher on the team prior to participating in the study to confirm their interest and eligibility. Participants were recruited based on the eligibility criteria and were required to provide informed consent and self-report that they had not used any EAP services before participating in the study and completing the survey.

Although recruitment was conducted through a large third-party research vendor with diverse participant panels, the sample should not be considered nationally representative of all workers in Canada or in the U.S.

### 2.4. Variables

This study examined cross-national differences of workers in their satisfaction with healthcare access, work ability, mental and physical job demands, overall quality of life (as measured using the WHOQOL-BREF), and self-reported happiness scores (as measured using the Oxford Happiness Questionnaire). The primary exposure was country of residence (Canada vs. the U.S.). Demographic and work-related characteristics, including income, education, age, gender, weekly work hours, work ability, and perceived job demands, were examined as potential predictors.

### 2.5. Data Collection

An 87-item questionnaire was developed based on previous occupational health studies and included both standardized instruments and original demographic questions. The survey included questions on a range of demographic factors, including gender, marital status, level of education, age, ethnicity, work experience (in years), and employment position. Additionally, standardized and validated measures that examine psychological well-being, quality of life, and working capacity were used in the survey. These measures included the Oxford Happiness Questionnaire (OHQ), the World Health Organization Quality of Life (WHOQOL-BREF) brief questionnaire, and the Work Ability Index (WAI). Data collection took place between September 2023 and April 2024 via REDCap,^29^ a web-based application for creating and distributing online surveys.

### 2.6. Outcome Measures

The 29-item Oxford Happiness Questionnaire (OHQ; derived from the Oxford Happiness Inventory [OHI]) assesses various dimensions of psychological well-being, including positive affect, life satisfaction, and personal fulfillment.^30^ Each item is presented as a single statement (e.g., “I feel that life is very rewarding”) and rated on a six-point Likert scale ranging from 1 (“Strongly Disagree”) to 6 (“Strongly Agree”). Negatively worded items are reverse coded so that higher scores reflect greater levels of happiness. The total happiness score is calculated by summing the scores across all items. Previous research has shown that all cross-scale correlations between corresponding OHQ and OHI items were highly significant (p < .001), and for most items, the correlations were strong.^30^ The OHQ has been demonstrated to possess adequate construct validity and internal reliability, as well as satisfactory structural validity.^31^

The WHOQOL-BREF is a shortened version of the World Health Organization Quality of Life Assessment (WHOQOL-100) designed to measure subjective quality of life across multiple life domains.^32^ The measure assesses four key areas: physical health, psychological well-being, social relationships, and environmental health. In addition to these domains, it includes two general items that evaluate overall quality of life and general health perception.^32^ Respondents rate each item using a five-point Likert scale, with scores ranging from 1 to 5. These raw scores are then converted to a 0-100 scale, where higher values indicate better quality of life.^33^ Past research has also demonstrated that the WHOQOL-BREF possesses robust cross-cultural reliability and construct validity.^33,34^

The Work Ability Index (WAI) is a self-report questionnaire designed to assess an individual’s perceived work capacity to their job demands and overall health status.^35^ It is commonly used in occupational health settings to determine a worker’s ability to perform their work tasks effectively. The WAI consists of seven dimensions: (1) current work ability compared with lifetime best, (2) work ability to the demands of the job, (3) number of current physician-diagnosed diseases, (4) estimated work impairment due to illness, (5) sick leave during the past 12 months, (6) personal prognosis of work ability two years from now, and (7) mental resources.^35^ Each dimension is assessed using a set of items, and the total unweighted score is calculated by summing across all seven dimensions, yielding scores ranging from 7 to 49.^35^ Higher scores reflect greater perceived work ability. Based on the total score, individuals are categorized into four standard work ability levels: poor (score of 27 or below), moderate (scores of 28–36), good (scores of 37–43), and excellent (scores of 44–49).^35^ The WAI showed satisfactory internal reliability, strong construct validity, and a high degree of cross-national stability.^36^

Overall, these three measurement instruments were selected for their strong psychometric properties, widespread international use, and appropriateness for assessing subjective well-being and occupational functioning across diverse populations.

### 2.7. Bias

To minimize potential bias, the study employed several safeguards during survey design, administration, and analysis. All measurement instruments were standardized, psychometrically validated, and administered identically across participants, which reduced measurement bias. Responses were anonymous and self-paced, which helped minimize social desirability bias. Selection bias was addressed by ensuring that eligibility criteria were strictly applied and by managing recruitment through a third-party vendor. However, the use of a non-random sample limits generalizability. Given the self-reported nature of all measures, there is also potential for common method variance. Missing data were addressed using multiple imputation. The imputation approach assumes that data were missing at random conditional on observed variables included in the imputation model.

### 2.8. Data Analysis

All statistical analyses were conducted using R (version 4.4.2) on macOS. A significance level of α = 0.05 was used, and tests were two-tailed unless otherwise specified. Descriptive statistics summarized the sociodemographic characteristics of the participants, as well as the distributions of quality of life and happiness scores. Missing data in key covariates were addressed using multiple imputation by predictive mean matching. Pooled estimates from the adjusted regression models were calculated using Rubin’s rules.^37,38^ Descriptive analyses and exploratory visualizations were based on the cleaned analytic dataset. The imputation approach assumes that missingness was missing at random conditional on observed variables included in the imputation model. Initially, we compared the scores of a range of variables across the two countries by standardizing the mean differences and then conducting t-tests. These comparisons included quality of life domains, happiness scores, income, work hours, and perceived job demands.

The central part of the analysis involved comparing overall quality-of-life and happiness scores across countries. This was done for the four domains in the WHOQOL scale (physical, psychological, environment and social). For the exploratory subgroup analysis, we compared quality of life (WHOQOL-BREF domains) and happiness (OHQ) between U.S. and Canadian respondents across several demographic and work-related categories. These included education level, ethnicity, income (classified as above or below the cohort median), and self-rated physical and mental work ability. To quantify the magnitude of differences between countries within each subgroup, we calculated the standardized mean difference (Cohen’s d). Precision of these estimates was assessed using 95% confidence intervals (CI). In accordance with standard conventions, effect sizes between -0.2 and 0.2 were considered negligible. Subgroup analyses were exploratory and intended to identify potential cross-national patterns across demographic and occupational characteristics rather than establish definitive causal or population-level conclusions.

For the second part of the analysis, we conducted country-stratified linear regression analyses to examine associations between demographic and work-related predictors and quality of life or happiness outcomes. Separate adjusted models were fitted for U.S. and Canadian respondents for each outcome variable, including WHOQOL physical, psychological, social, and environmental domains, OHQ score, satisfaction with living conditions, and satisfaction with health access. Predictors included age group, gender, income, education, work ability, tenure, physically demanding work, and retention intention. Regression estimates were pooled across imputed datasets using Rubin’s rules.^37^ Model fit statistics (R-squared, adjusted R-squared) and coefficient estimates with significance levels (p < 0.05) were compared between countries. We identified statistically significant predictors using a threshold of p < 0.05 with FDR adjustment for multiple comparisons. Multicollinearity and model diagnostics were assessed for each model to test the linear regression assumptions, with no violations and all VIFs below 5. A sensitivity power analysis was conducted using the observed country-specific sample size because an a priori power analysis was not conducted before recruitment. With 101 U.S. and 78 Canadian participants, the study had 80% power at α = 0.05 to detect an approximately moderate between-country difference, Cohen’s d = 0.42. Therefore, subgroup analyses were considered exploratory and interpreted cautiously.

## 3. Results

### 3.1. Demographic Analysis

The study included 179 participants, with 101 (56.0%) from the U.S. and 78 (44.0%) from Canada. Demographic and socioeconomic characteristics were compared between the two groups, with key differences observed in age and ethnicity (p < 0.05). Table 1 summarizes the distribution of variables, including gender, ethnicity, marital status, education, and economic indicators, along with their respective p-values for intergroup comparisons.

**Table 1.** Sociodemographic Characteristics of the Sample (n = 179)

Group	Variable	Overall (n=179)	United States (n=101)	Canada (n=78)	p-value
Gender	% Male	75 (41.9%)	48 (47.5%)	27 (34.6%)	0.135
	% Female	103 (57.5%)	52 (51.5%)	51 (65.4%)	​
	% Other Gender	1 (0.6%)	1 (1.0%)	0 (0.0%)	​
Ethnicity	% White	108 (60.3%)	52 (51.5%)	56 (71.8%)	0.009
	% Asian	25 (14.0%)	17 (16.8%)	8 (10.3%)	​
	% Black	31 (17.3%)	24 (23.8%)	7 (9.0%)	​
	% Other Ethnicity	15 (8.4%)	8 (7.9%)	7 (9.0%)	​
Marital Status	% Married	84 (46.9%)	47 (46.5%)	37 (47.4%)	0.378
	% Single	75 (41.9%)	45 (44.6%)	30 (38.5%)	​
	% Divorced	11 (6.1%)	4 (4.0%)	7 (9.0%)	​
	% Widowed	1 (0.6%)	1 (1.0%)	0 (0.0%)	​
Age Groups	Mean Age (SD)	38.9 (11)	36.4 (8.7)	42.3 (12.7)	<0.001
Education	% No High School	2 (1.1%)	0 (0.0%)	2 (2.6%)	0.543
	% High School	32 (17.9%)	18 (17.8%)	14 (17.9%)	​
	% College Certificate	20 (11.2%)	12 (11.9%)	8 (10.3%)	​
	% College Diploma	23 (12.8%)	12 (11.9%)	11 (14.1%)	​
	% Bachelor’s	51 (28.5%)	29 (28.7%)	22 (28.2%)	​
	% Master’s	47 (26.3%)	29 (28.7%)	18 (23.1%)	​
	% Other Education	4 (2.2%)	1 (1.0%)	3 (3.8%)	​
Economic	% Income <$20k	18 (10.1%)	6 (5.9%)	12 (15.4%)	0.425
	% Income $20-29k	15 (8.4%)	10 (9.9%)	5 (6.4%)	​
	% Income $30-39k	16 (8.9%)	9 (8.9%)	7 (9.0%)	​
	% Income $40-49k	14 (7.8%)	8 (7.9%)	6 (7.7%)	​
	% Income $50-59k	18 (10.1%)	10 (9.9%)	8 (10.3%)	​
	% Income $60-69k	13 (7.3%)	8 (7.9%)	5 (6.4%)	​
	% Income $70-79k	27 (15.1%)	19 (18.8%)	8 (10.3%)	​
	% Income $80k+	58 (32.4%)	31 (30.7%)	27 (34.6%)	​
Employment	Mean Work Hours (SD)	37.4 (10.7)	38.2 (11.7)	36.3 (9.3)	0.239
	Mean Tenure (SD)	7.8 (7.8)	7.1 (7.4)	8.6 (8.1)	0.222

*Note.* P-values indicate the results of t-tests or chi-squared tests, depending on whether the variable is numeric or categorical, respectively.

### 3.2. Overall Comparison

**Figure 2.**
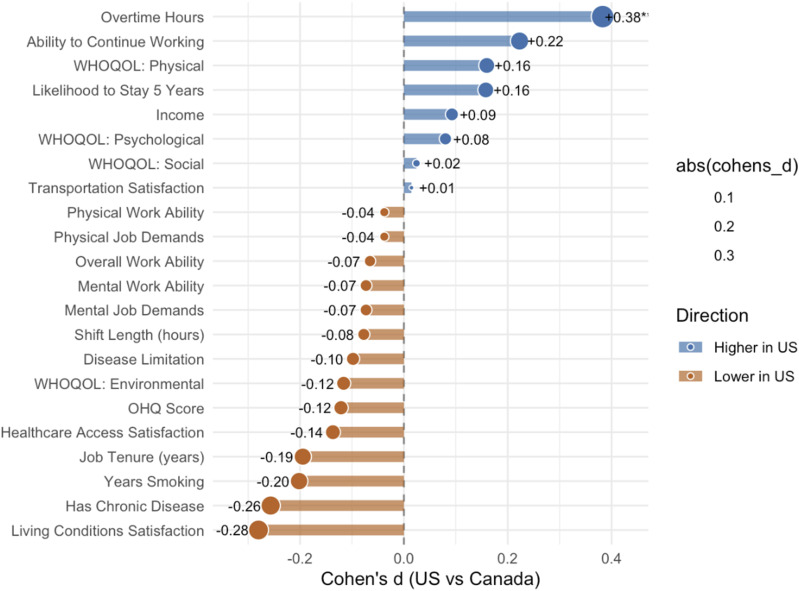
Standardized Mean Differences between Canada and the U.S. *Note.* Cohen’s d effect sizes are shown (0.2 = small, 0.5 = medium, 0.8 = large)

The standardized comparisons between U.S. and Canadian respondents revealed only a few notable differences in quality of life and occupational health measures (Figure 2). U.S. respondents reported higher overtime hours than Canadian respondents (M = 8.56 vs. 3.72; d = 0.38). Other standardized differences were small and non-significant, including income (M = 65,445.54 vs. 62,628.21; d = 0.09), work ability (M = 7.42 vs. 7.54; d = -0.07), physical quality of life (M = 65.66 vs. 61.44; d = 0.16), environmental quality of life (M = 66.98 vs. 69.92; d = -0.12), health access satisfaction (M = 3.61 vs. 3.76; d = -0.14), and living conditions satisfaction (M = 3.81 vs. 4.12; d = -0.28). The largest Canada-favouring standardized difference was observed for with living conditions (d = -0.28), although this comparison did not reach statistical significance. The effect sizes generally ranged from small to medium (Cohen’s d = 0.2-0.5), with overtime hours showing a moderate effect (d = 0.38), and it was the only statistically significant effect (p < 0.01). These patterns suggest that U.S. respondents reported higher overtime hours, while other observed country differences in income, work ability, physical quality of life, and environmental quality of life were small and not statistically significant.

Two-sample comparisons showed no significant overall country differences for WHOQOL physical (p = 0.301), social (p = 0.878), environmental (p = 0.455), or psychological (p = 0.624) domains. OHQ scores were also similar across countries (d = -0.12, p = 0.436). For the physical domain of the WHOQOL questionnaire, significant differences emerged among the 50- to 65-year-old age group (p adjusted = 0.036), where higher scores were observed for U.S. respondents. Moreover, U.S. respondents who had only completed a high school education also showed significantly higher physical QoL scores (p adjusted = 0.007) than their Canadian counterparts. Similarly, the social domain exhibited education-related disparities, with American high school diploma holders showing significantly higher scores (p adjusted < 0.001). Environmental quality of life varied slightly by income level, and no country differences were found for psychological health or occupational health scores.

### 3.3. Exploratory Subgroup Analysis

#### 3.3.1. Education and Socioeconomic Status

Respondents who completed high school in the U.S. reported significantly higher quality of life than their Canadian counterparts across multiple domains. This divergence was statistically significant in Social QoL (d = 2.06, CI 1 [1.11, 3.02]), Physical QoL (d = 1.73, CI [0.84, 2.61]), and Environmental QoL (d = 0.91, CI [0.08, 1.75]). Conversely, the U.S. advantage disappeared among higher-educated cohorts. Canadians with a University graduate degree reported statistically significantly higher Environmental QoL (d = -0.74, CI [-1.37, -0.10], while differences in other domains for this group were negligible.

Income-related differences were most pronounced for the below median salary group but non-significant. U.S. respondents reported higher Physical QoL (d = 0.33, CI [-0.09, 0.74]) and Social QoL (d = 0.24, CI [-0.18, 0.67]), though these specific point estimates did not reach statistical significance after accounting for variance.

#### 3.3.2. Ethnicity and Demographic Trends

Significant country-specific differences were observed within ethnic subgroups. Black respondents in Canada had numerically higher Social QoL and Environmental QoL than Black respondents in the U.S.; however, these subgroup estimates should be interpreted with caution due to small subgroup sizes and uncertainty. Respondents identifying with a mixed-race ethnicity in the U.S. reported higher scores of Physical QoL (d = 1.14, CI [-0.20, 2.49]) and Psychological QoL (d = 1.11, CI [-0.36, 2.58]) compared to Canadians of the same demographic, although these differences were not statistically significant.

#### 3.3.3. Work Ability Thresholds

Exploratory subgroup analyses suggested that country differences in quality-of-life outcomes may vary across self-reported work ability levels. Regarding mental work ability, Canadian respondents at a low-to-moderate level reported non-significant Psychological QoL (d=−0.77, CI [-2.64, 1.09]), whereas U.S. respondents at the highest level of ability reported non-significant Social QoL (d=0.53, CI [-0.15, 1.21]). Furthermore, at a high level of mental work ability, Canadian respondents reported numerically higher Environmental QoL, but this difference was not statistically significant (d = −0.50, CI [-1.02, 0.02]). Patterns in physical work ability also suggested possible divergence; at a low-to-moderate level, U.S. respondents reported numerically higher Happiness (d = 1.48, CI [-0.06, 3.03]) and Physical QoL (d = 0.73, CI [-0.68, 2.15]), but both confidence intervals crossed zero.

#### 3.3.4. Happiness (OHQ)

**Figure 3.**
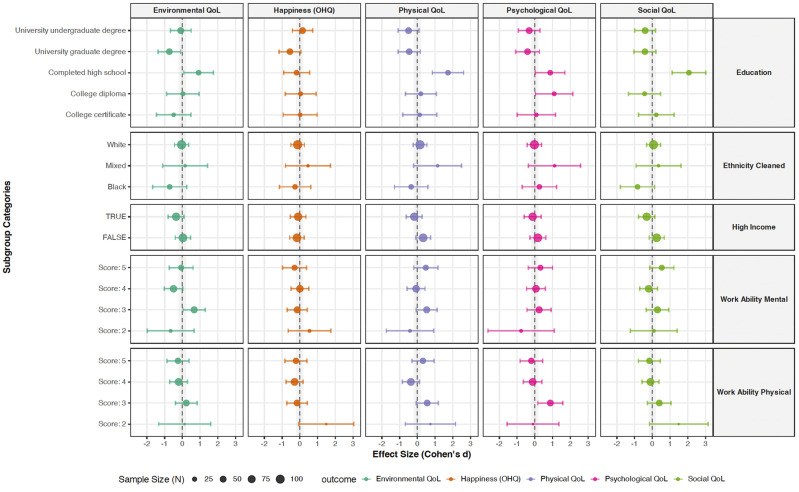
Comparative Analysis of Well-being Domains: US vs Canada *Note.* Standardized mean difference given by Cohen’s d, positive values indicate higher scores for U.S. respondents. In Figure 3, pooled multiple-imputation models were broadly consistent with the primary analysis, with work ability and income-related variables remaining important predictors across several outcomes. Full pooled estimates are reported in the Supplementary materials

Overall happiness scores remained remarkably consistent between countries across most subgroups. The only notable exception was among those with low physical work ability as noted above. For the majority of participants, including those grouped by White ethnicity (d = -0.13, p = 0.508) and High Income (d = -0.10, p = 0.667), the relationship between country of residence and subjective happiness was negligible (|d| < 0.2) and non-significant. Figure 3 presents the full comparative analysis of wellbeing domains between U.S. and Canadian populations. The full pooled estimates will be included in the Supplemental Materials.

## 4. Discussion

### 4.1. Summary

The purpose of the current study was to explore cross-national differences between Canadian and American workers who have never accessed EAP services, focusing on healthcare satisfaction, work demands, quality of life, happiness, and key demographic and employment characteristics.

The results showed no significant overall differences in quality of life or happiness between American and Canadian workers who had no prior experience of using EAP services. However, American participants reported significantly higher overtime hours. On the other hand, Canadian respondents had numerically higher work ability, physical health, and environmental quality-of-life scores though they were not statistically significant. Subgroup analyses revealed significant disparities by age, income, and education, with income having a stronger predictive value for well-being in the U.S., and work-related factors playing a greater role in Canada. Satisfaction with living conditions and access to healthcare also varied between countries, with different predictors emerging for each. Overall, the results suggest that economic factors may be more closely associated with well-being indicators in the U.S. sample, whereas occupational factors appeared more closely associated with well-being indicators in the Canadian sample. Given the cross-sectional design, modest sample size, and non-random sampling approach, the findings of this study should be interpreted as exploratory. The results are intended to identify potential patterns and associations rather than establish causal relationships.

### 4.2. Cross-National Patterns in Work and Well-Being

The current study found several notable differences across work and well-being contexts. First of all, American respondents reported significantly higher overtime hours and income compared to their Canadian counterparts. While recent peer-review literature that directly supported these findings is limited, the general consensus across several public sources suggests that workers in the U.S. work more hours annually compared to other OECD countries. For example, Wigender^39^ ranked the United States as the third highest country for hours worked. Earlier research explained that this discrepancy is likely due to cultural and economic differences. For example, Canadians were more likely to take extended vacations, work part-time, and have union coverage and labour standards compared to American workers,^40^ highlighting critical avenues through which Americans may be earning higher incomes than Canadians. More recent work shows that Canadians may have worked more hours on average per week.^38,41^ However, differences arise based on industry, demonstrating the need for further research.^42^

While these differences can have financial benefits for American workers, there may be potential tradeoffs regarding their physical and mental health. Existing literature has linked longer working hours with poorer occupational and mental health outcomes. Higher overtime hours among U.S. respondents may reflect broader labour-market and workplace differences between the two countries, but this study did not directly measure job insecurity, benefit access, or cost-of-living pressures. These findings may be interpreted in the context of broader differences between Canadian and U.S. healthcare and workplace systems, although this study did not directly test these structural mechanisms. Wong et al^43^ examined the effect of working hours on occupational health and found that long working hours is positively associated with sleep disturbance, short sleep duration, exhaustion, and injuries which have the strongest association. While not focused on American workers, other studies support the general finding that working overtime is associated with reduced mental health, including increased burnout and work-life conflict, reduced job satisfaction, irritability, fatigue, anxiety, depression and somatic responses.^44,45^

Though not significant, Canadian respondents endorsed higher levels in several well-being domains, including work ability, physical health, and environmental quality of life, relative to their American counterparts. Canadian respondents reported higher scores in several well-being domains; however, the present study did not directly examine structural or policy-level factors that may contribute to these differences. Several studies strongly support these findings,^46,47^ with an emphasis on how Canada’s redistributive social policies (e.g., unemployment benefits) and healthcare system support improved physical health in the long-term.^48^ Additionally, Frenette and Frank found that Canadians are more likely to have higher educational attainment, underscoring potential factors that support higher work ability and quality of life in Canadian workers.^49^ Despite potentially higher incomes, the absence of a significant difference in quality-of-life scores may reflect the added strain of private healthcare costs and economic pressures in the U.S., which could diminish the impact of earnings on well-being. These findings suggest that future research and workplace program development may benefit from considering country-specific patterns in overtime, work ability and quality of life.

### 4.3. Quality of Life Correlates

#### 4.3.1. Education and Socioeconomic Status

Education and socioeconomic status were key predictors of quality of life in respondents from both countries. U.S respondents who completed high school reported a higher quality of life compared to Canadian respondents, specifically in Social QoL, Physical QoL, and Environmental QoL domains. However, this effect disappeared among higher education cohorts in the Canadian context. This finding differs from recent work where higher education predicts higher quality of life compared to participants who had lower education.^50^ Interestingly, Canadians who graduated from university reported higher Environmental QoL, while differences in other domains remained small. A study by Tanguay et al suggested that factors such as higher median income influenced QoL within Canadian cities, due to being able to afford and support a better standard of living.^51,52^ Moreover, according to the OECD, higher educational attainment often leads to more earnings, as workers with upper secondary degrees earn around 18% more than non-degree holders in OECD countries.^52^ This suggests that Canadians’ high Environmental QoL in this study cohort may be due to increased access to better living conditions, as higher education can lead to a higher median income. However, small differences in other domains may be attributable to factors such as lower job satisfaction among non-degree holders. These interpretations remain speculative because the present study did not directly measure job-market competition, perceived return on education or career advancement opportunities.

#### 4.3.2. Ethnicity

This study also found country-specific differences within ethnic subgroups. Black respondents in Canada reported higher Social QoL and Environmental QoL scores than Black respondents in the U.S. Although the present study did not directly examine healthcare access, resilience, social support, or structural inequalities, prior literature has documented cross-national differences in social determinants of health and racial inequalities between Canada and the U.S.^51-53^ These findings highlight potential cross-national differences in quality-of-life outcomes within ethnic subgroups, though the underlying mechanisms were not examined in the present study. High Environmental QoL among Black Canadian respondents may be consistent with broader literature on healthcare access and social determinants of health that this study was not designed to evaluate. Boamah et al found that Black Canadians who had access to basic needs and healthcare had higher self-rated health (SRH) compared to those who had difficulties accessing healthcare.^53^ Several findings in the existing literature can help to explain why social quality of life may be greater among Black Canadians compared to Black Americans. For example, Boamah et al discuss that greater resilience among Black Canadians was linked to a higher quality of life.^53,54^ Furthermore, Ramraj et al conducted a cross-national study on differences in racial inequalities, finding greater Black-White inequalities in the U.S. compared to Canada.^55^ Overall, these findings seem to suggest stronger social networks and healthcare systems that support diverse ethnic groups in Canada compared to the U.S.

#### 4.3.3. Work Ability

The present study also found that work ability appeared associated with quality-of-life outcomes across several subgroup analyses. These findings should be interpreted with caution due to the exploratory nature of the analyses. In terms of mental work ability, Canadian respondents at low-to moderate levels reported higher Psychological QoL, whereas U.S respondents reported higher Social QoL. At a high level of mental work ability, Canadian respondents reported higher environmental QoL the U.S. respondents. This finding seems to be supported in the literature. For example, Meyer et al found that job autonomy and supervisor appreciation were linked to greater job satisfaction.^56^ These findings are consistent with the literature suggesting that perceived control over one’s work life may be associated with job satisfaction and work-related well-being. Physical work ability also showed a similar trend, where at low-to moderate levels, respondents in the U.S reported higher Happiness and Physical QoL compared to Canadians. This finding may be due to differences in workplace culture and accommodations between Canada and the United States. For example, several studies have shown that a sense of identity and fulfillment is strongly linked to one’s occupation.^57,58^ Accordingly, it stands to reason that greater ability to work might yield greater happiness and physical quality of life. Furthermore, cross-national differences in availability and usage of workplace accommodation may impact work ability, and subsequent happiness and quality of life.^59,60^

#### 4.3.4. Happiness

The present study revealed no significant difference in participants' happiness scores between Canada and the U.S. This finding seems to support existing literature, which shows that happiness is relatively stable across high-income countries, including Canada and the United States.^61,62^ However, it was observed that U.S respondents with low work physical ability, specifically those who scored at 2, reported higher happiness scores. Several studies have demonstrated a strong link between work demands and happiness, whereby higher demands predict lower happiness levels.^57,63,64^ One possible explanation is that workers with lower physical work ability may differ in job type, accommodations or work demands.^65,66^ However, these factors were not directly examined in the present analysis.

### 4.4. Limitations and Future Directions

Several limitations in the present study may have impacted the quality and generalizability of the findings. First, all data were collected through self-report, which is subject to various forms of response bias, such as social desirability and recall bias, thereby affecting response accuracy. The cross-sectional nature of the study also limits the ability to draw causal inferences and gain a deeper understanding of the underlying causal relationships. While the sample size was adequate for overall comparisons, it may have lacked sufficient statistical power for certain subgroup analyses. Additionally, the study focused exclusively on English-speaking participants, which may have excluded individuals with limited English proficiency who could have had different work and health experiences. The sample also lacked ethnic diversity, with only three groups (i.e., White, Black, and Asian) well-represented, limiting the applicability of the findings to more diverse populations. Furthermore, although the instruments used in the survey had previously demonstrated reliability and validity, the survey as a whole was not pilot tested in a specific population or context. As a result, the survey may have caused respondent fatigue and, in turn, lowered response rates.

To improve the generalizability of these findings across Canada and the U.S., future research should include a broader range of ethnic groups beyond Black, White, and Asian participants. Expanding representation will help ensure that the conclusions of this study better reflect the broader population. A related limitation is the aggregation of all Asian participants into a single category. This approach masks important within-group differences, as Asian populations are highly diverse in terms of culture, language, socioeconomic status, and health outcomes. Separating Asian populations into distinct subgroups by region (e.g., East Asian, West Asian, Central Asian, South Asian, Southeast Asian),^67,68^ should be considered in future research to capture within-group diversity within the Asian population. In addition, achieving a more balanced gender ratio in the sample is crucial for reducing potential biases and capturing a more comprehensive range of experiences. Further subgroup analyses are needed to identify distinct needs among different demographic groups, which can inform the development of more targeted and effective interventions. Moreover, in future studies, researchers could directly ask participants about the factors that affect their happiness levels via questionnaires or interviews to establish some degree of causality. Finally, based on the present findings, it is essential to evaluate whether existing EAPs are adequately equipped to address these unmet needs or if revisions are necessary to enhance their impact. The cross-sectional design and non-random sampling approach limit the ability to draw causal inferences. As such, the findings should be considered exploratory and hypothesis-generating, rather than confirmatory. Additionally, given how EAPs were not directly tested in this study, policy implications should be interpreted as suggestive rather than prescriptive.

## 5. Conclusion

This study examined differences in quality of life, happiness, demographic and work-related variables between Canadian and American workers with no experience of EAP services. While overall quality of life and happiness did not differ significantly by country, key subgroup differences emerged. U.S. participants reported higher overtime hours and income, while Canadian respondents reported higher work ability, physical health, and environmental quality of life. In the U.S., well-being was more strongly linked to income and education, whereas in Canada, work-related factors such as work ability had a greater impact. Findings of the present study suggest that future EAP research and program development may benefit from considering country and subgroup specific patterns in work ability, income, education, and quality of life. In particular, future studies may further explore whether workers with lower income or educational attainment in the U.S., as well as older workers or workers with reduced work capacity in Canada, experience distinct workplace support needs. Future research should include more diverse participants and examine whether current EAPs are effectively meeting these needs.

## Supplemental Material

Supplemental Material - Health, Well-Being, Work Ability and Work Conditions Among EAP Non-Users in Canada and the U.S.: A Quantitative Cross-National Comparison Study Supplemental Material for Health, Well-Being, Work Ability and Work Conditions Among EAP Non-Users in Canada and the U.S.: A Quantitative Cross-National Comparison Study by Hong Ki Chloe Lau, Javier Mencia Ledo, Kishana Balakrishnar, Sana Siddiqui, Raihana Premji, Paula Allen, Marilyn Grand'Maison, Allison Kelly, Ali Bani-Fatemi and Behdin Nowrouzi-Kia in Health Services Insights.

Supplemental Material - Health, Well-Being, Work Ability and Work Conditions Among EAP Non-Users in Canada and the U.S.: A Quantitative Cross-National Comparison Study Supplemental Material for Health, Well-Being, Work Ability and Work Conditions Among EAP Non-Users in Canada and the U.S.: A Quantitative Cross-National Comparison Study by Hong Ki Chloe Lau, Javier Mencia Ledo, Kishana Balakrishnar, Sana Siddiqui, Raihana Premji, Paula Allen, Marilyn Grand'Maison, Allison Kelly, Ali Bani-Fatemi and Behdin Nowrouzi-Kia in Health Services Insights.

Supplemental Material - Health, Well-Being, Work Ability and Work Conditions Among EAP Non-Users in Canada and the U.S.: A Quantitative Cross-National Comparison Study Supplemental Material for Health, Well-Being, Work Ability and Work Conditions Among EAP Non-Users in Canada and the U.S.: A Quantitative Cross-National Comparison Study by Hong Ki Chloe Lau, Javier Mencia Ledo, Kishana Balakrishnar, Sana Siddiqui, Raihana Premji, Paula Allen, Marilyn Grand'Maison, Allison Kelly, Ali Bani-Fatemi and Behdin Nowrouzi-Kia in Health Services Insights.
