# Supplementary material for: Health, Well-Being, Work Ability and Work Conditions Among EAP Non-Users in Canada and the U.S.: A Quantitative Cross-National Comparison Study
Source: Health Serv Insights. 2026 Jul 30;19:11786329261471267. doi: 10.1177/11786329261471267 (PMC13424505; doi:10.1177/11786329261471267)
Supplement: Supplemental Material - Health, Well-Being, Work Ability and Work Conditions Among EAP Non-Users in Canada and the U.S.: A Quantitative Cross-National Comparison Study [file sj-pdf-1-his-10.1177_11786329261471267.pdf]

Health Questionnaire

Please complete the survey below.

Thank you!

Occupational Science
& Occupational Therapy
UNIVERSITY OF TORONTO

Study Title: Exploring barriers and facilitators to the use of an Employee Assistance Program in Canada and the United States: A mixed-methods sequential exploratory study

Principal Investigator:
Behdin Nowrouzi-Kia, OT Reg. (Ont.), Ph.D., FRSA
Assistant professor
Department of Occupational Science and Occupational Therapy
Temerty Faculty of Medicine, U of T
E-mail: behdin.nowrouzi.kia@utoronto.ca Telephone: 416-946-3249

CONFLICT OF INTEREST STATEMENT
The researchers and staff in this study do not have any conflicts of interest, meaning we don't have any personal interests that could affect the study.

Principal Investigator
Dr. Behdin Nowrouzi-Kia is an occupational therapist and assistant professor in the Department of Occupational Science and Occupational Therapy in the Temerty Faculty of Medicine at the University of Toronto.

Study Sponsor
The study is funded by a Grant from the University of Toronto and TELUS Health.

Invitation to Participate
We invite you to join our study about using the Employee Assistance Program (EAP) in Canada and the United States. We want to learn about the things that help or make it hard for people to use the EAP. We'll also ask for your ideas to make it better.

Study Purpose
We're studying how people use the EAP for mental health support in North America. We want to find out what stops people from using it and how we can make it better.

Who Can Join
We want to hear from you if you've used an Employee Assistance Program in Canada or the United States.

What You'll Do
If you join, you'll answer questions in an online survey that takes about 25 minutes. You can also ask for a paper survey. The survey will ask you questions that evaluate the effectiveness of EAP services in improving your quality of life, happiness, and overall mental health. Your answers will help us understand how people use the EAP.

You Don't Have To Join
Your experiences are valuable and important to this study. It's up to you if you want to join. If you do, you can stop anytime without any problems. You can skip any question you don't want to answer. Your information is kept safe and private on the University of Toronto server.

What We'll Do With Your Answers
We will look at your answers to understand what people think. We'll share the results at meetings, write about them in a report, and provide de-identified interview transcripts to TELUS Health. TELUS Health will have access to de-identified data only, and your name or any personal details will not be included. Your name won't be shared, only a special number to keep your answers private. We'll keep your information safe for five years, then delete it.

Risks
Talking about work stress might make you feel upset. If that happens, you can stop anytime. You can also talk to the Employee & Family Assistance Program where you work, or your mental health provider if you have one.

Benefits
There's no direct benefit to you, but your answers will help us learn how to improve the EAP. If you want, we can send you a report about what we find.

Joining and Leaving the Study
You can join or leave the study whenever you want. Your work won't be affected. If you want us to delete your data, let Dr. Behdin Nowrouzi-Kia know. We'll keep the information collected until the study is done.

Costs and Payment
In recognition of your time, you will get $3 to $5 for your completion of the survey (Canadian dollars for Canadian participants and US dollars for participants from the United States).

It's Your Choice

You don't have to join the study. If you do, you can stop anytime. You can request to remove your information. If you have questions, just ask.

Confidentiality

We take your privacy seriously. All information about you will be kept private, following the law. Your rights are always protected, and signing this form doesn't change that. You'll be known only by a unique study ID number, not your name. The de-identified interview transcripts may be shared with TELUS Health, who will use this data to help improve the Employee Assistance Program (EAP). They must also keep your information confidential and will only receive data stripped of any personal identifiers.

The people in charge of this study, at the University of Toronto, are not doing this for any commercial gain. We might work with other research groups who want to use your health information for mental health research. They must also keep your information confidential.

Your data is stored safely on the University of Toronto server, and it's encrypted for extra security. If we share the results, your identity won't be revealed. All personal details are kept confidential in Dr. Nowrouzi-Kia's lab at 500 University Ave, Toronto, Ontario, Canada. We'll keep this data for five years to analyze, prepare reports, and decide if the project is feasible. After five years, we'll delete the de-identified data for your privacy.

Questions

For any questions about this study, please contact:

Behdin Nowrouzi-Kia, Ph.D., OT Reg. (Ont.)
Assistant professor,
Department of Occupational Science and Occupational Therapy, Temerty Faculty of Medicine, U of T E-mail:
behdin.nowrouzi.kia@utoronto.ca
Telephone: 416-946-3249

I have read the information presented in the Information Consent regarding the research projected conducted by Dr. Nowrouzi-Kia. I voluntarily consent to participate in this study. I am also aware that I can choose to withdraw my information within one week of receipt of the de-identified transcript or for a longer period upon my request.

Participant name: ______________________________

Date: ______________________________

Participant signature: ______________________________

Please indicate whether you consent to participating in this study.
- ◯ Yes
- ◯ No

Health Questionnaire

What is your gender?
- ◯ Female
- ◯ Male
- ◯ Transgender Female
- ◯ Transgender Male
- ◯ Gender Variant/Non-Conforming
- ◯ Not Listed
- ◯ Prefer Not to Answer

Not Listed, Please specify: ______________________________

What is your marital status?

- ◯ Single
- ◯ Married/Common-Law
- ◯ Separated
- ◯ Divorced
- ◯ Widowed

Please indicate the highest education you have obtained.

- ◯ Incomplete high school
- ◯ Completed high school
- ◯ College certificate
- ◯ College diploma
- ◯ University undergraduate degree
- ◯ University graduate degree
- ◯ Other please specify

Other, Please Specify:

____

What is your primary language?

____

What is your age as of your last birthday (in years)?

____

What is your ethnicity? (Check all that apply)

- ☐ Aboriginal
- ☐ East Asian
- ☐ South Asian
- ☐ West Asian
- ☐ South East Asian
- ☐ Black Caribbean
- ☐ Black African
- ☐ Black North American
- ☐ Indian Caribbean
- ☐ White European
- ☐ White North American
- ☐ Mixed Background
- ☐ Other
- ☐ Prefer Not to Answer

What is your height (feet and inches)?

____

What is your weight (in pounds)?

____

Which of the following would you use to describe yourself? (i.e., Tobacco use includes chewing smoking, or sniffing tobacco, and the use of products such as cigarettes, cigars, bidis, kreteks, and e-cigarettes)

- ◯ A daily tobacco user
- ◯ An occasional tobacco user
- ◯ A former daily or occasional tobacco user
- ◯ Non-smoker

If you are a current or former tobacco user, how many total years have you smoked? (Type N/A if not applicable) ____________________________________

How often do you have a drink containing alcohol?

- ◯ Never
- ◯ Monthly or less
- ◯ 2 to 4 times a month
- ◯ 2 to 3 times a week
- ◯ 4 or more times a week

How many drinks containing alcohol do you have on a typical day when you are drinking?

- ◯ 1 or 2
- ◯ 3 or 4
- ◯ 5 or 6
- ◯ 7 to 9
- ◯ 10 or more

Where were you born?

- ◯ Canada
- ◯ United States
- ◯ Other

Other, Please specify: ________________________________

Where do you currently live?

- ◯ Canada
- ◯ United States

How long have you been living in Canada (years)? ____________________________________

How long have you been living in the United States (years)? ____________________________________

Please indicate your total work experience in years. ____________________________________

Please indicate the average number of hours you work per week ____________________________________

Please indicate the average overtime hours you work per week ____________________________________

Do you intend to stay in your current position for the next 5 years?

- ◯ Yes
- ◯ No

How long have you worked at your current workplace in years?

____

Please indicate the usual duration of shift worked in your current position

- ◯ 4 hours
- ◯ 8 hour
- ◯ Other

Please specify the duration:

____

If No, please specify why

____

Which of the following best describes your gross annual income?

- ◯ Less than $20,000
- ◯ $20,000-$29,999
- ◯ $30,000-$39,999
- ◯ $40,000-$49,999
- ◯ $50,000-$59,999
- ◯ $60,000-$69,999
- ◯ $70,000-$79,999
- ◯ $80,000 or more
- ◯ Prefer Not to Answer

Below are a number of statements about happiness. Please indicate how much you agree or disagree with each by circling one of the options available. Please read the statements carefully; some of the questions are phrased positively and others negatively. Don't take too long over individual questions; there are no "right" or "wrong" answers (and no trick questions). The first answer that comes into your head is probably the right one for you. If you find some of the questions difficult, please give the answer that is true for you in general or for most of the time.

	Strongly Disagree (1)	Moderately Disagree (2)	Slightly Disagree (3)	Slightly Agree (4)	Moderately Agree (5)	Strongly Agree (6)
I don't feel particularly pleased with the way I am						
I am intensely interested in other people						
I feel that life is very rewarding						
I have very warm feelings towards almost everyone						
I rarely wake up feeling rested						
I am not particularly optimistic about the future						
I find most things amusing						
I am always committed and involved						
Life is good						
I don't think that the world is a good place						
I laugh a lot						
I am well satisfied about everything in my life						
I don't think I look attractive						
There is a gap between what I would like to do and what I have done						
I am very happy						
I find beauty in some things						
I always have a cheerful effect on others						
I can fit in (find time for) everything I want to						
I feel that I am not especially in control of my life						
I feel able to take anything on						
I feel fully mentally alert						
I often experience joy and						

elation I don't find it easy to make decisions

I don't have a particular sense of meaning and purpose in my life

I feel I have a great deal of energy

I usually have a good influence on events

I don't have fun with other
people

I don't feel particularly healthy

I don't have particularly happy memories of the past

Please read each question, assess your feelings, and circle the number on the scale that gives the best answer for you for each question.

	Very poor (1)	Poor (2)	Neither poor nor good (3)	Good (4)	Very good (5)
How would you rate your quality of life?	○	○	○	○	○

	Very dissatisfied (1)	Dissatisfied (2)	Neither satisfied nor dissatisfied (3)	Satisfied (4)	Very satisfied (5)
How satisfied are you with your health?	○	○	○	○	○

The following questions ask about how much you have experienced certain things in the last two weeks.

	Not at all (1)	A little (2)	A moderate amount (3)	Very much (4)	An extreme amount (5)
To what extent do you feel that physical pain prevents you from doing what you need to do?	○	○	○	○	○
How much do you need any medical treatment to function in your daily life?	○	○	○	○	○
How much do you enjoy life?	○	○	○	○	○
To what extent do you feel your life to be meaningful?	○	○	○	○	○

	Not at all (1)	Slightly (2)	A moderate amount (3)	Very much (4)	Extremely (5)
How well are you able to concentrate?	○	○	○	○	○
How safe do you feel in your daily life?	○	○	○	○	○
How healthy is your physical environment?	○	○	○	○	○

The following questions ask about how completely you experience or were able to do certain things in the last two weeks.

	Not at all (1)	A little (2)	Moderately (3)	Mostly (4)	Completely (5)
Do you have enough energy for everyday life?	○	○	○	○	○
Are you able to accept your bodily appearance?	○	○	○	○	○
Have you enough money to meet your needs?	○	○	○	○	○
How available to you is the information that you need in your day-to-day life?					
To what extent do you have the opportunity for leisure activities	○	○	○	○	○

	Very poor (1)	Poor (2)	Neither poor nor well (3)	Well (4)	Very well (5)
How well are you able to get around?	○	○	○	○	○

The following questions ask you to say how good or satisfied you have felt about various aspects of your life over the last two weeks.

	Very dissatisfied (1)	Dissatisfied (2)	Neither satisfied nor dissatisfied (3)	Satisfied (4)	Very satisfied (5)
How satisfied are you with your sleep?	○	○	○	○	○
How satisfied are you with your ability to perform your daily living activities?	○	○	○	○	○
How satisfied are you with your capacity for work?	○	○	○	○	○
How satisfied are you with yourself?	○	○	○	○	○
How satisfied are you with your personal relationships?	○	○	○	○	○
How satisfied are you with your sex life?	○	○	○	○	○
How satisfied are you with the support you get from your friends?	○	○	○	○	○
How satisfied are you with the conditions of your living place?	○	○	○	○	○
How satisfied are you with your access to health services?	○	○	○	○	○
How satisfied are you with your mode of transportation?	○	○	○	○	○

The follow question refers to how often you have felt or experienced certain things in the last two weeks.

	Never (1)	Seldom (2)	Quite often (3)	Very often (4)	Always (5)

How often do you have negative feelings, such as blue mood, despair, anxiety, depression? ○ ○ ○ ○ ○

The following questions refer to your work, your work ability and your health.

Is your workPsychologically demanding?

◯
◯
◯

Physically demanding?
Physically and psychologically demanding?

Current work ability compared to highest work ability ever:

	0	1	2	3	4	5	6	7	8	9	10
Assume that your work ability at its best has a value of 10 points. How many points would you give your current work ability? (0 means that you currently cannot work at all) (10 work ability at its best)	○	○	○	○	○	○	○	○	○	○	○

Work ability in relation to demands

	Very poor (1)	Rather poor (2)	Moderate (3)	Rather good (4)	Very good (5)
How do you rate your current work ability with respect to the physical demands of your work?	○	○	○	○	○
How do you rate your current work ability with respect to the mental demands of your work?	○	○	○	○	○

Current Diseases: In the following list, mark your current diseases or injuries. Also indicate whether a physician has diagnosed or treated these diseases.

No (0)

Injury due to an accident ○

Musculoskeletal disease in back, limbs or other part of the body (e.g. repeated pain in joint muscle, sciatica, rheumatism, arthritis) ○

Cardiovascular disease (e.g. ○hypertension, coronary heart disease)

Respiratory disease (e.g. ○repeated infections of the respiratory tract, emphysema)

Mental disorder (e.g. depression, ○"burn-out", anxiety or insomnia)

Neurological or sensory disease ○(e.g. hearing or visual disease, migraine, epilepsy)

Digestive disease / condition (e.g. gastritis, gall stones, liver or pancreatic disease, repeated constipation) ○

Genitourinary disease (e.g. ○infection in urinary tract, gynecological disease or prostate)

Skin disease (e.g. allergic or other rash, varicose veins) ○

Tumour or cancer ○

Endocrine or metabolic disease (e.g. diabetes, severe obesity or gout) ○

		Yes, physician's diagnosis (1)	Yes, own opinion (2)
Blood diseases (e.g. anemia, other blood disorder or defect)	○	○	○
Birth defects	○	○	○
Other disorder or disease	○	○	○

Is your illness or injury a hindrance to your current job? Check more than one alternative if needed.

- ☐ In my opinion I am entirely unable to work
- ☐ Because of my condition, I feel I am able to do only part time work
- ☐ I must often slow down my work pace or change my work methods
- ☐ I must sometimes slow down my work pace or change my work methods
- ☐ I am able to do my job, but it causes some symptoms
- ☐ There is no hindrance / I have no diseases

Illness within last year (12 months)

	100-354 days (1)	25-99 days (2)	10-24 days (3)	Max. 9 days (4)	None (5)
During the last 12 months, how many whole days have you been off work because of illness	○	○	○	○	○

Estimation of own work ability in 2 years

	Unlikely (1)	Not Certain (4)	Relatively certain (7)
Do you believe, according to your present state of health, that you will be able to do your current job two years from now?	○	○	○

Mental capacities

	Never (0)	Rather seldom (1)	Sometimes (2)	Rather often (3)	Often (4)
Considering the last three months, have you been able to enjoy your regular daily activities?	◯	◯	◯	◯	◯
Considering the last three months, have you been active and alert?	◯	◯	◯	◯	◯
Considering the last three months, have you felt yourself to be full of hope about the future?	◯	◯	◯	◯	◯
