# Supplementary material for: Health, Well-Being, Work Ability and Work Conditions Among EAP Non-Users in Canada and the U.S.: A Quantitative Cross-National Comparison Study
Source: Health Serv Insights. 2026 Jul 30;19:11786329261471267. doi: 10.1177/11786329261471267 (PMC13424505; doi:10.1177/11786329261471267)
Supplement: Supplemental Material - Health, Well-Being, Work Ability and Work Conditions Among EAP Non-Users in Canada and the U.S.: A Quantitative Cross-National Comparison Study [file sj-pdf-2-his-10.1177_11786329261471267.pdf]

**Country**	**Outcome**	**Predictor**	**Beta**	**95% CI**	**p-value**
U.S.	WHOQOL physical	Income $50,000-$59,999	29.07	7.68, 50.46	0.008
U.S.	WHOQOL physical	Income $60,000-$69,999	24.95	1.97, 47.92	0.034
U.S.	WHOQOL physical	Income $70,000-$79,999	30.56	8.56, 52.56	0.007
U.S.	WHOQOL physical	Income $80,000 or more	24.39	3.73, 45.05	0.021
U.S.	WHOQOL physical	Work ability	5.94	3.06, 8.83	<0.001
Canada	WHOQOL physical	Work ability	6.58	3.64, 9.52	<0.001
Canada	WHOQOL psychological	Income $70,000-$79,999	38.12	6.82, 69.42	0.018
Canada	WHOQOL psychological	Physically demanding work	33.62	1.42, 65.82	0.041
U.S.	WHOQOL social	Education: Other specify	-73.38	-135.17, -11.59	0.021
U.S.	WHOQOL social	Work ability	3.92	0.26, 7.58	0.036
Canada	WHOQOL social	Completed high school	-30.84	-56.96, -4.72	0.022
U.S.	WHOQOL environmental	Education: Other specify	-52.13	-103.38, -0.89	0.046
U.S.	WHOQOL environmental	Work ability	3.51	0.47, 6.55	0.024
Canada	WHOQOL environmental	Income $30,000-$39,999	35.31	1.86, 68.77	0.039
Canada	WHOQOL environmental	Income $50,000-$59,999	41.71	11.23, 72.19	0.008
Canada	WHOQOL environmental	Income $60,000-$69,999	49.27	14.16, 84.39	0.007
Canada	WHOQOL environmental	Income $70,000-$79,999	47.08	13.55, 80.61	0.007
Canada	WHOQOL environmental	Income $80,000 or more	40.26	9.86, 70.67	0.011
Canada	WHOQOL environmental	Income less than $20,000	46.18	18.20, 74.16	0.002
U.S.	OHQ score	Completed high school	-0.55	-1.05, -0.05	0.030
U.S.	OHQ score	Tenure	-0.02	-0.04, -0.003	0.020
U.S.	Living conditions satisfaction	Education: Other specify	-2.67	-5.15, -0.19	0.035
Canada	Living conditions	Income $50,000-$59,999	1.66	0.30, 3.02	0.018

Country	Outcome	Predictor	Beta	95% CI	p-value
	satisfaction				
Canada	Living conditions satisfaction	Income $60,000-$69,999	2.11	0.53, 3.68	0.010
Canada	Living conditions satisfaction	Income $70,000-$79,999	1.94	0.43, 3.45	0.013
Canada	Living conditions satisfaction	Income $80,000 or more	1.80	0.43, 3.17	0.011
Canada	Living conditions satisfaction	Income less than $20,000	2.48	1.19, 3.77	<0.001
Canada	Living conditions satisfaction	Work ability	0.18	0.004, 0.35	0.045
U.S.	Health access satisfaction	Work ability	0.18	0.03, 0.33	0.021
Canada	Health access satisfaction	Income $30,000-$39,999	2.12	0.57, 3.66	0.009
Canada	Health access satisfaction	Income $40,000-$49,999	2.04	0.30, 3.77	0.023
Canada	Health access satisfaction	Income $50,000-$59,999	1.86	0.37, 3.34	0.016
Canada	Health access satisfaction	Income $60,000-$69,999	2.16	0.49, 3.82	0.013
Canada	Health access satisfaction	Income $70,000-$79,999	2.11	0.48, 3.74	0.013
Canada	Health access satisfaction	Income $80,000 or more	1.79	0.34, 3.24	0.017
Canada	Health access satisfaction	Income less than $20,000	2.04	0.71, 3.38	0.004
Canada	Health access satisfaction	Physically demanding work	1.72	0.07, 3.36	0.041
